# Causes and Associated Factors of Headaches among 5 to 15-year-old Children Referred to a Neurology Clinic in Kashan, Iran 

**Published:** 2015

**Authors:** Ahmad TALEBIAN, Babak SOLTANI, Mostafa HAJI REZAEI

**Affiliations:** 1 Department of Pediatrics, Kashan University of Medical Sciences, Kashan, Iran; 2 Trauma Research Center, Kashan University of Medical Sciences, Kashan, Iran

**Keywords:** Migraine, Tension-type headache, Children, Prevalence

## Abstract

**Objective:**

Headaches are common neurologic problems for children and adolescents. They are divided into two types: primary and secondary. Primary headaches include migraines and tension-type as well as comprise the majority of headaches. We detect the causes of headaches and their associations with demographic variables among children and adolescents.

**Materials & Methods:**

This cross-sectional study was performed on 5–15 year-old children with headaches from March 2010 to April 2012 who presented at a pediatric neurology clinic in Kashan, Iran. Diagnosis of headaches was done in accordance with the International Classification of Headache Disorders. Data regarding the type of headache, age, gender, pain severity, aura, family history, and sleep disorder were collected.

**Results:**

One hundred fourteen children (44 male and 70 female) with headaches were enrolled in the study. The types of headaches were comprised as follows: 67 cases of migraines, 38 cases of tension-type headaches, 2 cases of cluster headaches, and 7 cases of secondary headaches. Pulsating headaches, family history of headaches, insomnia, and pain severity had higher prevalence in migrainous patients.

**Conclusion:**

Physicians should extend their information gathering about primary and secondary headaches. Sleep disturbances and a family history of headaches were the most important factors associated with migraine headaches.

## Introduction

Headaches are a common problem in children and adolescents and are a major cause of school absenteeism and health-associated costs (1). The prevalence of recurrent and severe attack of headaches increases with age incrementally from 4.5% among 4–6 year-old children to 27.4% among adolescents of 16–18 years of age (2). The headache prevalence among males and females is the same before 12 years of age (almost 10%) but increased in girls after 12 years of age (28–36% versus 20% for boys) (2, 3). Children with a lower socioeconomic status had a higher prevalence of headaches (4). Headaches in children and adolescents may be classified into primary (migraine, tension-type, and cluster headaches) or secondary (upper respiratory infections, central nervous system infections, or space occupying lesions) etiologies (5). Primary headaches comprise most of headaches in children and adolescents (6). Cluster headaches are severe and usually unilateral (periorbital) and last less than three hours. They are usually associated with ipsilateral lacrimation, rhinorrhea, ophthalmic injection, and (sometimes) Horner syndrome (7). The most common cause of acute recurrent childhood headaches are migraines (7). Some studies have detected a history of atopy, a family history of headaches, and a low socioeconomic status were more prevalent in cases with migraines (8). Sleep disturbances are more prevalent in children with migraines. Children and adolescents with migraines have poorer sleep quality and greater difficulty falling asleep, night awakenings, daytime sleepiness, and nocturnal symptoms, such as sweating, sleep talking, bruxism, sleepwalking, and nightmares, than the headache-free control group did. In adolescents with migraines, the frequency and intensity of the headache as well as duration of the headache history were related to occurrences of sleep disturbances (9-11). 

The importance of childhood headaches and other reports about their etiologies and associated factors, we decided to determine the causes and some associated variables and compare them with other investigations. 

## Materials & Methods

In a cross-sectional study conducted between March 2010 and April 2012 and following written informed consent from the parents of test subjects between 5–15 years-of age. A total of 114 children were enrolled. They were referred to the pediatric neurology clinic of Kashan University of Medical Sciences in Kashan, Iran with either a diagnosis of primary or secondary headache. 

The patients were examined by a pediatric neurologists and imaging was performed if indicated (12). Inclusion criteria were included all previously healthy 5–15 year-old-children with headaches, normal psychomotor development, and cooperative parents. Diagnosis of tension-type and migraine headaches was according to the International Classification of Headache Disorders, 2nd edition (ICHD-II) (7). Collected data included: age, sex, type of headache, family history of headache, duration of headache, location of headache (unilateral or bilateral), quality of headache (pulsating or non-pulsating), sleep disorders (insomnia or oversleep), aura, and pain severity scale (0–10 scale) based on the Headache Intake Questionnaire and other clinical signs and symptoms associated with headaches such as nausea, vomiting, photophobia, and phonophobia (14). 

All data recorded in questionnaires and statistical analysis was performed with SPSS version 16 for Windows. Quantitative variables were indicated as mean ± SD and qualitative variables were shown as numbers and percentages. Descriptive statistics, Chi square, T test, and Fisher exact test were used for statistical analysis. P-values were two-tailed and p<0.05 was considered significant. 

## Results

One hundred fourteen 5-15 year-old-children referred to Kashan pediatric neurology clinic were evaluated. Totally, the mean age was 8.88±2.74. Forty-four (38.6%) children were male and 70(61.4%) were female. The mean age in males and females was 8.52±2.7 and 9.1±2.76 respectively. There was no significant age difference between both sexes (P=0.27). 

The mean age of cases with migraine was 9.25±2.72 and tension headache was 8.51±2.68, the difference between them was not statistically significant (P=0.181). 

Causes of headaches comprised of 67 (58.8%) cases of migraines, 38 (33.3%) cases of tension-type headaches, 2 (1.8%) cases of cluster headaches, and 7 (6.1%) cases of secondary headaches. Secondary headaches included head trauma (3 patients), cerebrovascular accident (CVA) (2 patients), brain abscess (1 patient), and brain tumor (1 case). According to the high frequency of migraine and tension-type headaches as the primary headache group in our study, these patients were evaluated statistically and more comprehensively. Pulsating headaches were more prevalent in children with migraines rather than tension-type headaches (P<0.001, OR=36.81, CI: 8.1-167). Aura was present in 22.4% of patients with migraines. There was no association between aura with age (P=0.78) and gender (P=0.49). A family history of headache was significantly higher in cases of migraines compared to tension-type headaches (65.7% versus 34.2%) (P=0.002, OR=3.67, CI: 1.59-8.51). Furthermore, a family history of headaches was positive in 63.6% of males versus 44.3% of females (P=0.044, OR=2.2, CI: 1.01-4.77). Nausea and vomiting were seen in 26.9% of patients with migraine versus 5.3% of tension-type headaches (P=0.007, OR=6.6, CI: 1.44-30.32). Insomnia was reported more often in migraines (55.2%) rather than for the tension-type headache group (26.3%) (P=0.004, OR=3.45, CI: 1.45-8.22). The mean pain severity scale in the migraine group was 6.28±1.2 versus 5.53±1.1 in the tension-type group and was significantly different (P=0.002, CI: 0.29-1.22). No significant association was detected between types of headaches and some variables such as age, location of headaches (unilateral or bilateral), gender, duration of headaches (≤24 hours versus >24 hours), oversleep, photophobia, and phonophobia (Table 1). 

## Discussion

This study evaluated the causes of childhood headaches with ICHD-II criteria (7). Our investigation detected no association between primary headaches (migraine, tension-type headache) and age in spite of Donald’s results, which indicated an increase of headache frequency with age (15). In the present study, gender was not a factor in any age group and primary headache types were in contrast to Kroner-Herwig et al, which declared an increase of primary headaches among females (16). Sex differences in childhood headaches was documented in some surveys (17) and in contrast to our study. Among girls, the stress of social limitations, social expectations, and more respect for boys in families can increase the prevalence of headaches (18). In Ayatollahi’s report (19), the prevalence of headache increased by age and tension-type headaches were significantly more likely in girls, which is also incompatible with our study. In some studies, the prevalence of migraines was greater in males (4,20). The frequency of migraines in our patients was greater than tension-type headaches, which is in contrast with the Kroner-Herwig study (16). Our findings determined a high frequency of migraines in children with a positive history of primary headaches in their parents. It is comparable with reports from Fallahzadeh et al (20) and Kroner-Herwig (16), which indicated an association of a family history of headaches with childhood migraines when compared to tension-type headaches. Russel MB et al revealed that a positive family history of tension-type headaches in first-degree relatives was not concordant with our study in which there was no association between tension type and a family history of headache (21). Additionally, in this study, a family history of headaches was more positive in males than in females. The rate of headache family history in some investigations was about 80% (9, 10) while it was 51.8% in our study. There was strong association between migraine headaches and insomnia in this study (odds ratio=3.45) that is concordant with Miller at al r (11). Some studies have shown an association of childhood headaches with oversleep (10, 11), which is incongruent with our research. Miller et al reported high prevalences of sleep disorders in children with migraines (11). Isik et al (10) demonstrated an association of migraine with parasomnia (teeth grinding, sleep verbalization, nightmares, bedtime struggle, and sleep walking). According to Stewart et al (22), the peak age of migraines without aura among female children was 14–17 years of age; while present study indicated there was no association between aura with age and gender. Ayatollahi et al has shown a significant association between type of headaches (migraine and tension-type) and duration of pain (hours), quality of headaches (pulsating, nonpulsating), intensity of pain, and location of headaches (unilateral, bilateral) but no association was detected with nausea, vomiting, photophobia, and phonophobia (19). In our research, there was a marked association between type of headaches (migraine and tension-type) with the duration of pain, quality of headache, intensity of pain, nausea, and vomiting while there was no association with location of headache, photophobia, and phonophobia. In our research, due to a small number of cluster and secondary headache cases, we can indicate descriptive statistics and analyze statistically the association of some variables with migraines and tension-type headaches. The main limitation of our study is small sample size of secondary and cluster headache cases. Furthermore, the socioeconomic status of patients and psychological factors (family or school problems) affecting headaches was not evaluated in the present study. Therefore, further investigations with a larger sample size to provide more comprehensive data for associated factors that influence childhood headaches are proposed. 

**Table 1:** Characteristics compared between cases of migraine and tension type headaches

Variables		Migraine No (%)	Tension No (%)	p-value	OR	CI
Age (years)	5–10	46(68.7%)	27(71.1%)	0.798	0.892	0.37-2.1
	10<	21(31.3%)	11(28.9%)			
Location of pain	Unilateral	23(34.3%)	11(28.9%)	0.571	1.283	0.54-3.04
	Bilateral	44(65.7%)	27(71.1%)			
Gender	Male	29(43.3%)	12(31.6%)	0.237	1.65	0.72-3.82
	female	38(56.7%)	26(68.4%)			
Duration of headache (hours)	≤24	58(86.6%)	36(94.7%)	0.32	0.358	0.07-1.75
	>24	9(13.4%)	2(5.3%)			
Photophobia	No	40(59.7%)	22(57.9%)	0.856	1.07	0.48-2.41
	Yes	27(40.3%)	16(42.1%)			
Phonophobia	No	40(59.7)	21(55.3%)	0.658	1.19	0.54-2.68
	Yes	27(40.3%)	17(44.7%)			
Oversleep	No	59(88.1%)	32(84.2%)	0.577	1.38	0.44-4.3
	Yes	8(11.9%)	6(15.8%)			

OR= Odds Ratio, CI= Confidence Interval

In conclusion, headaches are a common and debilitating problem in children and adolescents. Physicians require more knowledge regarding primary and secondary headaches and their associated factors in children and adolescents. An exact history, physical, and neurologic examinations are prudent for diagnosis of headache type. A family history of headaches and sleep disorders were the most significantly associated factors with migraine headaches. 

## Notes

**Funding Support: **Kashan University of Medical Sciences.
